# The Banded One-Anastomosis Gastric Bypass Trial (RiMini Trial): Protocol of a Prospective Single-Center Randomized Controlled Trial

**DOI:** 10.1007/s11695-025-07751-6

**Published:** 2025-03-14

**Authors:** Maureen Tissink, Tim Verhagen, Ian Faneyte, Eric Hazebroek, Sake Oost, Josien Timmerman, Anouk Veldhuis, Marc van Det

**Affiliations:** 1 Hospital Group Twente, Almelo, Netherlands; 2 Rijnstate Hospital, Arnhem, Netherlands

**Keywords:** Severe obesity, Obesity, Banded one-anastomosis gastric bypass, One-anastomosis gastric bypass, OAGB, Weight loss, Metabolic bariatric surgery, MBS, %TWL

## Abstract

**Background:**

Long-term recurrent weight gain remains a persistent challenge in metabolic bariatric surgery (MBS). One strategy for managing recurrent weight gain involves the placement of a non-adjustable silicone ring around the reduced stomach pouch. This technique may lead to more significant weight loss and a reduced risk of long-term recurrent weight gain. Although several studies have demonstrated the effectiveness of silicone rings in combination with Roux-en-Y gastric bypass (RYGB), randomized studies providing long-term data on the effectiveness of primary banded one-anastomosis gastric bypass (OAGB) are lacking.

**Methods:**

A total of 210 patients will be included in this prospective, non-blinded, single-center randomized controlled trial. The primary endpoint is the difference in total weight loss percentage (%TWL) 5 years post-surgery. Secondary outcomes include excess weight loss percentage (%EWL), changes in obesity complications, quality of life, and adverse events related to the surgical procedures. The study population will consist of patients eligible for primary OAGB aged 18 years and older.

**Conclusions:**

The RiMini trial aims to investigate whether there is a significant difference in long-term weight reduction expressed as %TWL in patients undergoing an OAGB with or without the addition of a silicone ring 5 years after surgery.

**Trial Registration:**

This study is registered at Clinicaltrials.gov (NCT05472922) on the 25th of July, 2022.

**Graphical Abstract:**

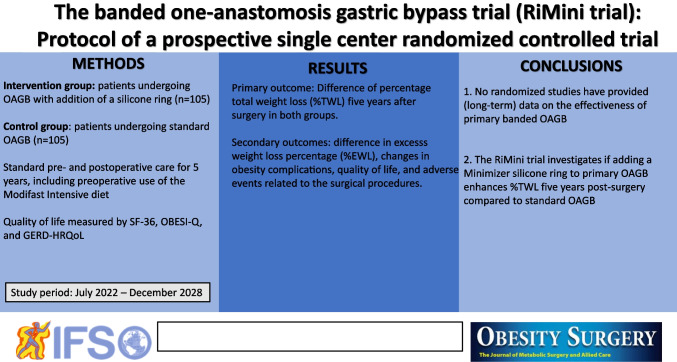

## Introduction

Overweight and obesity have reached epidemic proportions worldwide, contributing to an array of chronic health conditions significantly impacting overall public health. In the Netherlands, this global challenge is also evident, with approximately 50% of adults being overweight, 16% suffering from obesity, and 4% classified as severely obese [1]. Obesity is associated with an elevated risk of complications such as diabetes mellitus, hypertension, obstructive sleep apnea syndrome (OSAS), osteoarthritis, gastroesophageal reflux disease (GERD), and dyslipidemia. Obesity reduces life expectancy by 8 to 10 years, similar to smoking, and is a major avoidable cause of cancer, responsible for one in seven cancer deaths in men and one in five in women [2, 3]. Given the severe health consequences of obesity, metabolic bariatric surgery (MBS) has emerged as a crucial treatment option and is considered the most optimal treatment for significant weight loss and reduction or remission of obesity complications [4].

However, recurrent weight gain after initial weight loss remains a persistent challenge. Defining the prevalence of significant recurrent weight gain is complicated due to various factors such as patient characteristics, anatomic variations, surgical techniques, follow-up periods, and, most importantly, the lack of a standardized definition for recurrent weight gain [5–7]. Approximately 50% of post-bariatric surgery patients experience recurrent weight gain of at least 5% of their initial body weight within the first 2 years after surgery [8]. Estimates for more significant recurrent weight gain range from 20 to 30% [9], although some studies with small sample sizes have reported even higher rates [6].

Recently, the International Federation for the Surgery of Obesity and Metabolic Disorders (IFSO) updated their statement on the effectiveness of the OAGB as a weight loss procedure [10]. They stated that the OAGB is at least equivalent to RYGB and potentially superior to sleeve gastrectomy (SG) and gastric banding (GB). OAGB has also been promoted by some as a technically easier and safer alternative to the RYGB [11].

As with other bariatric procedures, patients can struggle with recurrent weight gain after initially optimal OAGB [12]. As mentioned before, addressing weight gain is a complex and multifaceted challenge. In some cases, revisional MBS is considered an option, but some of these revisional procedures are associated with a considerably high risk of complications and often yield unsatisfactory outcomes [9]. The most commonly known revisional procedures are conversion from the gastric band or sleeve to a gastric bypass, limb lengthening of a gastric bypass, or pouch revision including revision of the gastro-jejunal anastomosis. A new alternative to the abovementioned revisional procedures involves longitudinal reduction of the pouch (or sleeve) and placement of a non-adjustable silicone ring around the gastric pouch. This technique may also lead to more significant weight loss and a reduced risk of long-term recurrent weight gain [13, 14]. Given the challenges posed by recurrent weight gain, it seems more reasonable to pursue all possible ways of reducing the risk of recurrent weight gain at the time of the primary procedure.

While studies have suggested the effectiveness of silicone rings in combination with primary RYGB, there is a lack of data on their use with primary OAGB [14]. Although the OAGB and RYGB share similar mechanisms of action, they differ in certain anatomical aspects such as pouch dimensions. Traditionally, the RYGB with a small pouch is considered to have a more restrictive nature, while the OAGB, with its longer and narrower pouch, is thought to be more hypo-absorptive. However, recent studies have introduced successful modifications to the RYGB, such as a longer pouch and an extended biliopancreatic limb, which resemble some of the characteristics of the OAGB [15]. Conversely, the addition of a silicone ring to the OAGB may enhance restriction, addressing a potential limitation in the original procedure. To date, no randomized studies have provided (long-term) data on the effectiveness of primary banded OAGB.

The RiMini trial aims to investigate whether incorporating a silicone ring during primary OAGB improves weight reduction, measured as %TWL 5 years after surgery, compared to classic OAGB. Secondary endpoints include changes in obesity complications, quality of life, and adverse events related to surgical procedures.

## Methods

### Study Design and Patient Selection

This study is a single-center, non-blinded, prospective randomized controlled trial. The intervention group consists of patients undergoing standard OAGB with the addition of a MiniMizer silicone Ring (Bariatric Solutions GMBH, CH-8260 Stein am Rhein), placed 2 to 4 cm below the esophagogastric junction (see the “Surgical Techniques” section). The control group consists of patients undergoing standard OAGB without the addition of the silicone ring. All procedures will be performed by one of five bariatric surgeons. Of these, two surgeons will exclusively perform standard OAGB in the control group, while the other three surgeons will conduct both the intervention and standard procedures.

Patients meeting the general (inter)national criteria for MBS [4] are potentially eligible for participating in this study. Exclusion criteria are shown in Table 1. Patients are informed about the study during their preoperative screening. Participation is voluntary, and patients are offered a 2-week period to consider their decision before signing the informed consent form.

**Table 1:** Exclusion criteria

Previous MBS
Insufficient proficiency in the Dutch language
Genetic conditions affecting compliance with medical advice
Chronic inflammatory bowel diseases
Renal dysfunction (eGFR < 30) or liver dysfunction (ASAT/ALAT twice the normal values)
Pregnancy before or during the study
Clinically severe therapy-resistant GERD
Silicone allergy

After providing informed consent, patients are randomly assigned to one of the two groups using a computer-generated randomization sequence to ensure balanced allocation.

All patients will receive standard multidisciplinary pre- and postoperative care, in accordance with the local standards of care. This includes preoperative blood tests and the “Modifast Intensive®” diet for 2 weeks, providing 850 kcal/day to reduce liver volume, promote weight loss, and improve surgical conditions and recovery [16]. Postoperative care is provided for 4 months, followed by annual evaluations for up to 5 years, including blood tests and assessment of obesity complications. In this study, patients will also complete standardized questionnaires in addition to the standard care (see the “Quality of Life” section).

### Quality of Life

Participating patients are asked to complete three questionnaires pre- and postoperatively during the follow-up period, the SF-36, OBESI-Q, and GERD-HRQoL, to assess quality of life.

The SF-36 questionnaire includes 36 items and measures eight health concept scales: Physical Functioning, Role-Physical, Bodily Pain, General Health, Vitality, Social Functioning, Role-Emotional, and Mental Health. The raw scores of each subscale are transformed to a 0–100 scale, where higher scores indicate better functioning or well-being. A minimum of 50% of the items in each subscale must be answered to yield a viable result [17].

The OBESI-Q questionnaire includes a set of six selected subscales from the BODY-Q that have been individually validated for Dutch practice [18, 19]. The BODY-Q measures four overarching domains. Each domain includes four or more independently functioning scales. Scales chosen in OBESI-Q include eating behavior, social, psychological, physical function, body image, and sexual. Scores for each outcome are calculated by summing up the points for each subscale. To enhance the interpretability of the sum score, it is converted to a total score on a scale of 0 to 100, where higher scores indicate better functioning or well-being.

The GERD-HRQoL questionnaire includes 18 items to measure the impact of GERD on sleep, exercise, diet, need for medication, sex life, work, social activity, and psychological well-being [20]. The total score is calculated by summing up the individual item scores, with no transformation of the raw scores into a scaled score required. The optimal score is 0, which indicates that the individual is asymptomatic across all categories, while the lowest possible score is 50, indicating complete incapacitation in all categories.

### Surgical Techniques

Standard surgical techniques are used in all study patients. A long and narrow pouch is formed from the angle of the lesser curvature to the angle of His. Calibration of pouch diameter is done loosely around a 34 fr nasogastric tube. A standard-length biliary limb of 150 cm for patients with BMI up to 50 kg/m^2^ or 180 cm for patients with BMI above 50 kg/m^2^ is drawn cranially in an antecolic antegastric position. A 30/40-mm stapled side-to-side gastro-jejunostomy is created with a linear endostapler. The remaining defect is closed with a 23-cm 3–0 absorbable V-loc running suture, starting the suture more proximally on the biliary limb and aligning this along the longitudinal stapling line of the pouch. In the intervention group, a silicone ring is placed 2 to 4 cm caudal to the gastro-esophageal junction. The chosen circumference of the silicone ring is 7.5 cm (approximately 24 mm internal diameter). The silicone ring is secured to the longitudinal staple line with a non-absorbable suture. Control of the integrity of the anastomosis and staple line is performed in both groups by air leak testing after the introduction of a gastric tube by the anesthesiologist. Additional sutures are placed in case of leakage.

The silicone ring is a CE-marked medical device by Bariatric Solutions, which consists of a silicone-coated blunt tip and a ring body with four locking positions. Once the ring is secured, the blunt tip will be cut off and removed.

### Sample Size Calculation

In order to obtain mid-term results, a 5-year follow-up period has been selected, consistent with standard practices in Dutch metabolic bariatric surgery. A total of 63 patients per group is necessary to detect a 5% difference %TWL between groups 5 years post-surgery. This calculation is performed using PASS software, with parameters set at power = 80%, alpha = 0.05, standard deviation = 10%, and average %TWL of group 1 vs. group 2 being 32 vs. 27, assuming a two-sided independent *t*-test.

Jense et al. observed a loss-to-follow-up (LtfU) ranging from 21 to 58% three to five years postoperatively [21]. To have sufficient power for the 5-year analysis, this study takes into account a LtFU of 40%, assuming that actively contacting patients will reduce LtFU at 5 years postoperatively. Based on a required sample of 63 individuals per group and an expected LtFU of 40%, we aim to include 210 patients for this study, 105 per group.

## Outcome Measurement

All outcomes are measured at 4 months and annually up to 5 years postoperatively.

### Primary Outcome

%TWL is defined as the percentage of weight loss relative to the total body weight at the time of informed consent [22]. In formula

$${\%}TWL=\frac{\text{preoperative weight}-\text{ postoperative followup weight}}{\text{preoperative weight}}\text{x}\;100$$

### Secondary Outcomes

1. %EWL: defined as the percentage of excess weight lost relative to the excess weight at the time of informed consent [22]. In formula
  $${\%EWL}=\frac{\text{preoperative BMI}-\text{ current BMI}}{\text{preoperative BMI}-25}\text{x}\;100$$
2. Reduction or remission of obesity complications: Obesity complications include type 2 diabetes mellitus, hypertension, joint complaints, dyslipidemia, OSAS, and GERD. (Inter)national guidelines are used as reference [23, 24]. Reduction is defined as a decrease in symptoms, medication use and/or dosage, and/or laboratory results. Remission is defined as the state in which a patient no longer reports symptoms, does not require any medication for their comorbidity, and/or has normalized laboratory results. In case of type 2 diabetes, remission is defined if glycosylated hemoglobin (HbA1c) is 43 mmol/mol without the use of pharmacotherapy as per local protocol [23] In case of OSAS, remission is considered when there is a discontinuation of CPAP treatment.
3. Quality of life: assessed using the SF-36, OBESI-Q, and GERD-HQoL questionnaires at earlier mentioned follow-up intervals compared to pre-operative baseline measurements.
4. Presence of early and late dumping: determined based on symptoms reported by patients and evaluated by expert physicians during the follow-up period.
5. Complications: All complications during follow-up and until 5 years postoperative are included in the analysis, regardless of their potential relation to the ring. Complications are categorized as perioperative, early (< 30 days, with or without readmission), and late (> 30 days, with or without readmission). Possible complications include bleeding, wound infections, intra-abdominal abscesses, anastomotic leaks, deficiencies (especially in vitamin B12 and ferritin), conversions, migration, erosion, and slipping of the ring. Laboratory reference values are used for deficiencies.

### Statistical Analysis

IBM’s Statistical Package for the Social Sciences (SPSS) is utilized for the statistical analyses of the obtained data. Histograms (visual inspection) are used to ascertain whether variables are normally distributed. A significance level (alpha) of 0.05 is applied to all analyses unless stated otherwise. Differences in baseline characteristics of the study groups are tested using the independent *t*-test (for continuous, normally distributed data), the Mann–Whitney test (for non-normally distributed or ordinal data), or the chi-square test (for categorical data).

Assumptions for linear regression will be checked for all outcomes, including normality of residuals and homogeneity of variances. Where necessary, transformations (e.g., log transformation) were applied to meet model assumptions.

Difference between groups in %TWL, %EWL, and GERD-HRQoL at 5 years postoperative will be assessed using the independent *t*-test or Mann–Whitney test (for normally or non-normally distributed data, respectively). Baseline characteristics related to both the type of surgery and outcome variables (with *p*-value < 0.15) will be considered confounders in the analyses. If one or more confounders are identified, linear regression models will be used to assess differences between groups in %TWL, %EWL, and GERD-HRQoL at 5 years postoperative, each adjusted for relevant confounders.

The difference between groups in the reduction of obesity complications at 5 years postoperative will be tested using chi-square tests for each comorbidity. Holm-Bonferroni correction will be used to correct for multiple testing.

Chi-square tests will be used for the difference between groups in change of scores of the questionnaires, early and late dumping, and reflux. Additionally, a linear mixed model analysis will test the difference in change in the subscales of the SF-36 and OBESI-Q over time between groups.

The difference in the total number of complications between groups will be tested with independent *t*-tests or Mann–Whitney tests (for normally or non-normally distributed data, respectively).

Missing data will be reported if known. A sensitivity analysis will be performed for the primary outcome: analysis of the data “as-is” (without imputation) will be compared with the results of the analysis where missing data is imputed.

## Data Availability

No datasets were generated or analysed during the current study.

## Declarations

### Trial Status

The trial is currently running. The protocol used is the 2nd version submitted to the ethical committee board (April 2022). The recruitment started in July 2022 and finished in December 2023.
